# A Manual of Surgical Treatment

**Published:** 1903-09

**Authors:** 


### BOOK REVIEWS.

A Manual of Surgical Treatment. By W. Watson Cheyne, M. B., F. R. C. S., F. R. S., Professor of Surgery in King's College, London. And F. F. Burghard, M. D. and M. S. (Lond.), F. R. C. S., Teacher of Practical Surgery in King's College, London. Volume VII. The treatment of the surgical affections of the rectum, the liver, pancreas and spleen, the genitourinary organs, the breast and the thorax. Philadelphia and New York: Lea Brothers & Co. 1903. (Price, $5.75.)

The long awaited last volume of Cheyne and Burghard's surgery has been issued. The size of the volume and the amount of material it contains are sufficient excuse for the delay in appearance. In reviews of previous volumes, the general scope and method of procedure have been very clearly described and there remains but little to say on these points relative to this volume, save that the plan originally laid down for the conduct of the work has been closely adhered to, and operative procedures are outlined with the same clearness and the same simplicity of construction which marked the descriptive work all through the series.

The present volume includes the surgical affections of the rectum and anus in which is given a very comprehensive description of the regional anatomy as a basis of the work described; the surgical affections of the spleen, liver and pancreas; the surgical affections of the breast and thorax, together with affections of the thorax and its contents.

The greater part of the volume is given up to surgical affections of the genitourinary organs and this constitutes the only blemish of the entire work, viewed from an American standpoint. The anatomy of the section is excellently set forth; but the balance of the department is weak, in some places misleading and in others absolutely useless. This last is best illustrated by a reference to organic stricture, which is discussed at considerable length. In the treatment dilatation is suggested and a metal bougie is advocated; the diagnostic procedure is very carefully illustrated and the pictures show the passing of sounds, which are evidently what are meant by "metal bougies;" there can be no error in this; the fact is patent that a strictly therapeutic instrument is advocated for purely diagnostic purposes; and the use of flexible bougies-a-boule, or acorn-tipped, as they are termed in the book, is frowned upon.

This is a most amazing stand on the part of the authors, who are conceded to be and have shown themselves excellent surgeons. Their frank admission that much of the genitourinary material has been taken from Morris, confirms the suspicion that they are rather weak on genitourinary procedures and while their frankness is commendable they have shown an anxiety too apparent to advocate procedures which are not up-to-date and are not good surgery. They have committed the error of giving much space to matters with which they are not thoroughly familiar and have slighted others which they might have amplified with very little research.

In the pages devoted to the surgery of the prostate much stress is laid on the suprapubic route in prostatectomy and the work of only two American surgeons is mentioned, and then very briefly, Alexander and Parker Syms (spelled Sims in the book); so brief that one might say the reference was merely fleeting. No word of support is given to the perineal route which is conceded to be the best method and the late J. P. Bryson, of Saint Louis, acknowledged to be one of the world's masters of prostatectomy, is not even mentioned,—even by suspicion. Cheyne and Burghard believe in cutting along the floor of the urethra in dividing a stricture, even a deep one; they even go further by stating unequivocally that stricture, spasmodic or organic, is never found in the prostatic urethra,—a fact which has been disproved by demonstration on more than one occasion.

Gonorrhea in the female is dismissed in 21 lines of type, aggregating a little over 200 words, telling absolutely nothing. Male gonorrhea is given more prominence without adding anything to the subject in the way of treatment, except a rather guarded advocacy of what we may consider one of the high crimes in genitourinary work,—the passing of a catheter in acute cases. Irrigation is not advocated. It is regrettable that genitourinary surgery, one of the most important branches, should have been so poorly handled. There can be no possible excuse for it for, certainly, the authors of the work had access to American textbooks, Fuller, White and Martin and to Syms's papers and Bryson's, any of which would have given them material and statistics sufficient to lead them into the rosy paths of correct surgical literature. These authors have made the mistake which many writers of genitourinary textbooks have made, even on this side of the water, of jumping at conclusions. They can hardly be blamed seriously though, when one considers that of all the textbooks on the subject which have been published in this country there are not three which may be properly considered of valuable use to either the practitioner or the student. There has yet to be written the ideal textbook. Eugene Fuller has come nearer to it than anyone else.

However, in spite of the shortcoming mentioned in the one particular instance of genitourinary surgery, Cheyne and Burghard have written a very excellent work on general surgery, which is well-constructed and of material aid in operative procedures and after-treatment. N. W. W.

